# First-year students’ math anxiety predicts STEM avoidance and underperformance throughout university, independently of math ability

**DOI:** 10.1038/s41539-021-00095-7

**Published:** 2021-06-14

**Authors:** Richard J. Daker, Sylvia U. Gattas, H. Moriah Sokolowski, Adam E. Green, Ian M. Lyons

**Affiliations:** 1 Department of Psychology, Georgetown University, Washington, D.C., United States; 2 Department of Experimental Psychology, University of Oxford, Oxford, United Kingdom; 3 Rotman Research Institute, Baycrest Health Sciences, Toronto, Canada

**Keywords:** Psychology, Education

## Abstract

Math anxiety is widely considered a potential barrier to success in STEM. Current thinking holds that math anxiety is directly linked to avoidance of and underperformance in STEM domains. However, past evidence supporting these claims is limited in important ways. Perhaps most crucially, it is possible that math anxiety predicts STEM outcomes merely as a proxy for poor math skills. Here, we tested the link between math anxiety and subsequent STEM outcomes by measuring math anxiety, math ability, and several covariates in 183 first-semester university students. We then tracked students’ STEM avoidance and achievement through four years at university via official academic transcripts. Results showed that math anxiety predicted both a reduction in how many STEM courses students took and, separately (i.e., controlling for one another), lower STEM grades. Crucially, these associations held after controlling for math ability (and other covariates). That math anxiety predicts math-related academic achievement independently of Math Ability suggests that, contrary to current thinking, math anxiety’s effects on academic performance likely operate via mechanisms other than negatively affecting math ability. Beyond this, we show evidence that math anxiety can account for associations between math ability and STEM outcomes, suggesting that past links between math ability and real-world outcomes may, in fact, be at least partially explainable by attitudes toward math. These findings provide clear impetus for developing and testing interventions that target math anxiety specifically and suggest that focusing on math ability without additional attention to math anxiety may fail to optimally boost STEM outcomes.

## Introduction

An important goal of researchers, policy-makers, and educators is to foster engagement and success in science, technology, engineering, and mathematics (STEM), especially at the university level where specialized STEM training often begins. Researchers and educators alike believe that anxiety specific to math, or math anxiety, acts as an important barrier to both STEM participation and achievement^1–3^. This is a powerful idea because if it is correct, this suggests that fulfilling the promise in STEM is not just a matter of one’s cognitive ability but also depends on one’s emotions. The idea that math anxiety acts as a barrier to STEM outcomes has important practical implications as well—if anxiety toward math plays an active role in holding students back from succeeding in STEM, then educational interventions aiming to boost STEM outcomes should target math anxiety. Theory on math anxiety holds that math anxiety has two key negative consequences: (1) avoidance of math and (2) underperformance in math^4^. When applied to academic situations, both of these theorized consequences might plausibly affect students’ prospects of success in STEM—if math anxiety causes students to avoid coursework in STEM and/or to underperform in STEM courses, this would stifle students’ ability to be successful in STEM fields. However, the evidence supporting these putative negative academic consequences of math anxiety is limited in important ways that constrain both our theoretical understanding of the potential negative effects of math anxiety on academic STEM outcomes and our ability to make recommendations on the development of targeted interventions to boost STEM outcomes. Below, we briefly summarize the evidence supporting links between math anxiety and these theorized negative outcomes before turning to a discussion of crucial limitations in this body of evidence.

Highly math-anxious individuals are thought to avoid math whenever possible^1,4^. The evidence supporting this stems largely from work showing that individuals higher in math anxiety tend to report having taken fewer high school and university-level math courses than their less math-anxious counterparts^5^. Meece, Wigfield, and Eccles^6^ also showed that math anxious individuals report being less likely to take additional math courses in the future. Another source of evidence for a link between math anxiety and math avoidance has focused on decisions to engage in STEM, examining the relative math anxiety levels of students in STEM vs. non-STEM majors and of adults in STEM vs. non-STEM careers. Individuals in non-STEM majors and in non-STEM careers report significantly higher levels of math anxiety than those in STEM majors^5,7^ and careers^8^, respectively. Longitudinal work by Ahmed^9^ found that twelfth-grade high school students who had either been consistently high in math anxiety since middle school or who had become highly math-anxious over time were less likely to hold a STEM career as adults than students who either had consistently low levels of math anxiety from middle school through high school or who had become less math-anxious since middle school. Math anxiety is also thought to negatively affect performance in math-related courses^4,10^. Previous studies have shown that math anxiety is associated with poorer performance in high school and university-level math classes^5^. Math anxiety has also been shown to negatively predict grades earned in psychology methods and statistics course^11^.

The work reviewed above provides some evidence to suggest that math anxiety may lead students to avoid STEM courses when possible and to underperform in STEM courses that they cannot avoid. However, the body of evidence supporting math anxiety as a barrier to participation and achievement in STEM is limited in important ways, and several key theoretical questions have not yet been tested.

One crucial limitation is that nearly every previous study supporting a link between math anxiety and STEM academic outcomes (with the exception of work by LeFevre, Kulak, and Hemans^7^) did not account for the fact that high math anxiety and poor math ability are robustly linked^12,13^. Previously observed links between math anxiety and STEM outcomes (both avoidance of STEM and underperformance in STEM) could therefore be explained by shared associations with math ability. This is a critical weakness in the evidence base linking math anxiety to STEM outcomes, especially if the goal is to use research to inform educational interventions. If math anxiety is merely a proxy for poor math skills when predicting STEM outcomes, then interventions to boost STEM outcomes might do better to simply focus on improving math skills. But if math anxiety predicts STEM outcomes over and above math skills, this would suggest interventions that ignore math anxiety may miss an opportunity to effectively increase STEM participation and achievement.

Addressing whether math anxiety predicts STEM outcomes even when controlling for math ability would also inform our understanding of the mechanisms that link math anxiety with potential negative academic consequences. First considering avoidance of math as a consequence of math anxiety, it is fairly straightforward to hypothesize that math anxiety would predict *avoidance* of math-related courses over and above math ability—when students are making decisions about what courses to choose, for instance, their attitudes toward math are likely to matter just as much as, if not more than, their objective ability in math. Directly testing this hypothesis would nevertheless provide valuable evidence as to whether math anxiety is relevant in shaping decisions to pursue or avoid STEM even when individual differences in Math Ability are accounted for.

In a similar vein, asking whether math anxiety predicts grades earned in STEM courses over and above math ability may provide a more nuanced understanding of the mechanisms by which math anxiety relates to poor STEM *achievement*. There are two main mechanisms by which math anxiety is thought to lead to poor performance in math-related courses. One account focuses on previous avoidance of math: if students consistently avoid math courses, over time, they are likely to gain less practice with math, thereby stunting the development of their math ability^1,4^. The other account focuses on the in-the-moment effects of math anxiety on math performance. For students who are high in math anxiety, the prospect of having to do math is anxiety-inducing. This online anxiety response is thought to co-opt working memory resources that are necessary for doing difficult math, thereby causing math-anxious students to underperform on working memory-demanding math tests^4,14^. In both of these explanations, math anxiety is related to academic performance via its influence on math ability, either in the past by leading to a reduction in experience with math, or in the moment by taking up working memory resources. If differences in math ability were found to explain associations between math anxiety and STEM achievement, this would provide support for these kinds of models of the academic consequences of math anxiety. If, on the other hand, math anxiety predicted individual differences in STEM performance over and above math ability, this would suggest that math anxiety and STEM outcomes are associated through mechanisms that are not dependent on math ability, pointing to a need to update theories by which math anxiety is related to academic outcomes.

A simple way to overcome this limitation of previous work and to address these theoretical questions would be to measure math ability alongside math anxiety and assess whether math anxiety continues to predict unique variance in STEM outcomes when math ability is controlled for. Doing so would inform theory on math anxiety by allowing for an understanding of the extent to which the predictive effects of math anxiety on STEM avoidance and underperformance can be explained by math ability. Interestingly, the reverse is true as well. A great deal of research has linked math ability with important life outcomes, including grades in math courses^15^, decisions to pursue STEM careers^16–18^, and even income levels and health outcomes^19,20^. None of this previous research, however, has accounted for differences in math anxiety. Just as it is possible that math ability may have confounded previously observed relations between math anxiety and STEM outcomes, the reverse is also true: Prior reports of relations between maths ability and STEM outcomes may have been inflated by failing to control for anxiety about math. By including both types of math measures, one can test the extent to which each *uniquely* predicts STEM outcomes (i.e., controlling for one another).

A second limitation concern claims that math anxiety has negative consequences for STEM outcomes broadly considered^1–3^. The issue is that studies supporting a link between math anxiety and academic outcomes have largely focused specifically on math outcomes rather than on STEM outcomes more broadly. For instance, existing evidence examining the relation between math anxiety and STEM *achievement* has focused either on a handful of math courses^5^ or on a single psychology research methods course^11^. This is not necessarily a flaw of the individual studies themselves (it is perfectly reasonable to assess the effects of math anxiety on math outcomes), but it does limit confidence that math anxiety has negative consequences for STEM outcomes more broadly. No research we are aware of has assessed whether math anxiety predicts performance in STEM courses in a manner that includes all sub-areas of STEM (namely, science, technology, engineering, and math).

Furthermore, while there are a small number of studies showing that math anxiety correlates with *avoidance* of STEM majors or careers^5,7–9^, the majority of these studies were in fact retrodictive. The measure of math anxiety was collected *after* the STEM-related behavior in question (e.g., math anxiety was collected after individuals had declared a STEM major or attained a STEM career). From both a practical and a theoretical standpoint, it would be preferable to establish the *predictive* validity of math anxiety—namely, does math anxiety predict subsequent STEM outcomes? The lone study that measured math anxiety prior to the STEM outcome in question (STEM career choice^9^) is limited by the absence of control for math ability, as discussed above. (Conversely, the lone study that controlled for math ability^7^ was retrodictive.) Addressing these gaps requires is a single study that collects measures of math anxiety at the outset—for instance, as students matriculated at university—and predicts subsequent STEM outcomes, while also controlling for objective math ability.

A third limitation of previous work linking math anxiety to STEM outcomes is that STEM avoidance and STEM achievement are often conflated. That is, no single study to our knowledge has linked math anxiety with both STEM avoidance and STEM achievement. As a result, the two types of STEM outcomes are often used interchangeably when discussing the implications of math anxiety for STEM. However, whether one chooses to take courses in STEM (or not), and how well one does in those STEM courses are not necessarily the same thing. Are these two measures so highly correlated that using one as a proxy for another is not in fact all that problematic? Alternatively, if these two aspects of STEM relatively uncorrelated, one can then ask whether math anxiety is primarily predictive of taking fewer STEM courses (STEM avoidance), how well one does in the courses one does take (STEM achievement), or both. If in fact Math Anxiety independently predicts one or both types of STEM outcomes (i.e., controlling for one another), this would suggest that both of the theorized consequences of math anxiety—avoidance and poor performance—may operate via independent mechanisms. This in turn can potentially enrich our theoretical understanding by pointing to specific routes through which math anxiety is linked to negative STEM outcomes. On a practical level, answering these questions may also help shape expectations about how interventions aimed at curbing math anxiety might realistically impact specific STEM outcomes. The present study aims to fill this gap in present understanding by collecting measures of both STEM participation (or the lack thereof) and STEM achievement in the same dataset.

We contend that if we are to take seriously the idea that math anxiety is a key leverage point for researchers and educators interested in fostering better university STEM outcomes, more direct evidence that overcomes the limitations of previous work is needed. We, therefore, measured math anxiety among first-semester university students and predicted real-world university STEM participation and STEM achievement over the ensuing four years of university. Importantly, we assessed the extent to which math anxiety predicted each of these STEM outcomes, even when controlling for one another, for individual differences in math ability, and several other covariates. This allowed us to ask whether anxiety toward math and objective ability in math both predict unique variance in STEM participation and achievement. Further, rather than relying on student self-reports of academic outcomes, we used actual university transcripts to tabulate STEM outcomes. Transcripts provided a comprehensive account of the extent to which students participated in STEM courses and their level of achievement in those courses throughout the university. Together, this design allowed us to test long-standing assumptions in the literature about the role of math anxiety in real-world STEM outcomes, and to establish greater specificity in terms of the scope and limitations of this role.

We also leveraged this design to address two secondary questions. First, we sought to use a mediation approach to directly quantify the extent to which associations between math anxiety and STEM outcomes can be accounted for by individual differences in math ability. We also used a similar framework to test the more counterintuitive idea that prior reports of relations between maths ability and STEM outcomes may in fact be explained—at least in part—by math anxiety. Second, we asked whether math anxiety was especially predictive of university STEM outcomes for certain types of students. math anxiety tends to be higher in women on average^5,21^, and it tends to most strongly affect performance among those who tend to be higher in general cognitive ability^22–24^. Thus, we tested whether the link between math anxiety and STEM outcomes depended on (1) Gender and (2) non-STEM achievement levels.

## Results

### Relations between math measures and between STEM outcomes

Before addressing the main theoretical question of the extent to which first-semester math anxiety predicts university-level STEM outcomes, we first asked some more basic questions of this sample. First, we tested the extent to which Math Anxiety and Math Ability were related in the present sample. Results showed that Math Ability and Math Anxiety were negatively correlated [*r*(181) = −.346, *p* = 2E − 6; this and all other statistical tests presented are two-sided]. This negative relation and its magnitude are consistent with a large body of the previous research^5,10,12,13^, suggesting that these measures are operating as expected in the present sample.

Second, we tested the extent to which % STEM Courses and STEM Grades were associated with one another. Perhaps surprisingly, we found no significant relationship between the two [*r*(181) = .119, *p* = .107]. University students who do not intend to ultimately pursue STEM disciplines, however, often take STEM courses in their first semester or two to satisfy general education requirements. Students who left the university early may therefore have inflated estimates of the % STEM Courses they would have taken throughout the normal course of a four-year degree. When the partial correlation between % STEM Courses and STEM Grades controlling for Semesters Absent was computed, we found that there was a significant relation between % STEM Courses and STEM Grades [partial-*r*-value, *r*_*p*_(180) = .191, *p* = .010], and the zero-order relation between % STEM Courses and STEM Grades among those with no Semesters Absent was also significant [*r*(146) = .163, *p* = .047]. However, even the highest of these estimates suggests that less than 5% of the variance in one STEM outcome can be explained by the other. This provides evidence that STEM participation and STEM achievement are distinct, which underscores the importance of examining % STEM Courses and STEM Grades as separate outcome measures.

For descriptive statistics and zero-order correlations between all measures, including all covariates, see Table S2.

### Zero-order relations between first-semester Math Anxiety and Math Ability and university STEM outcomes

We next assessed whether Math Anxiety and Math Ability predicted % STEM Courses and STEM Grades without controlling for any covariates. Zero-order correlations showed that Math Anxiety and Math Ability were each predictive of individual differences in both % STEM Courses and STEM Grades (all *p*s ≤ .001). The observed pattern of associations—Math Anxiety and Math Ability both predict differences in STEM participation and achievement and are themselves significantly associated—strongly underscores the need to control for Math Ability when assessing whether Math Anxiety predicts STEM outcomes. Any observed relations between Math Anxiety and STEM outcomes could very well be driven by shared associations with Math Ability if differences in Math Ability are not accounted for.

In the native units of each STEM outcome measure, an increase of one standard deviation in Math Anxiety was associated with a 13.1% decrease in % STEM Courses [*r*(181) = −0.390, *p* = 5E − 8] and a 3.47 point drop in STEM Grades [*r*(181) = −0.324, *p* = 8E − 6]. An increase of one standard deviation in Math Ability was associated with an 8.1% increase in % STEM Courses [*r*(181) = 0.241, *p* = 0.001] and a 3.00 point boost in STEM Grades [*r*(181) = 0.280, *p* = 1E − 4]. Note that controlling for Semesters Absent does not substantially affect these relations: [Math Anxiety and % STEM Courses *r*_*p*_(180) = −0.396, *p* = 3E − 8; Math Anxiety and STEM Grades *r*_*p*_(180) = −0.339, *p* = 3E − 6; Math Ability and % STEM Courses *r*_*p*_(180) = 0.282, *p* = 1E − 4; Math Ability and STEM Grades *r*_*p*_(180) = 0.272, *p* = 2E − 4].

### Unique predictive effects of first-semester Math Anxiety and Math Ability on four-year STEM outcomes

In this section, we assessed the extent to which students’ math anxiety and math ability during the first semester of university *uniquely* predicted university STEM participation and achievement over four years at university, when controlling for one another and for other relevant cognitive, affective, and academic variables.

**Fig. 1:**
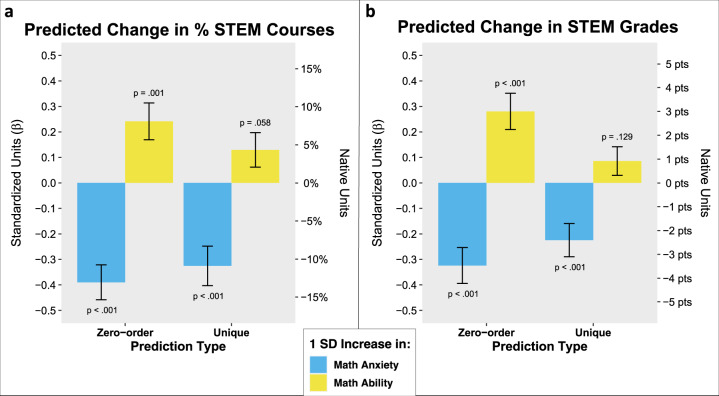
Zero-order and unique predictive effects of first-semester Math Anxiety and Math Ability on university STEM outcomes. Figure **a** shows the change in % STEM Courses associated with a 1 SD (standard deviation) increase in Math Anxiety and Math Ability. Zero-order relations between each predictor and % STEM Courses are plotted alongside unique relations between each predictor and % STEM Courses predicted by a multiple regression model including the following measures as predictors: Math Anxiety, Math Ability, STEM Grades, Trait Anxiety, Verbal Working Memory, Gender, non-STEM Grades, and Semesters Absent. Figure **b** is the same as Fig. **a** but displays the change in STEM Grades (in points out of 100) associated with a 1 SD increase in Math Anxiety and Math Ability as the dependent variable. The multiple regression model that generated the unique predictions included the same measures as predictors as that of Fig. **a**, but substituted % STEM Courses for STEM Grades. In both figures, the left *y*-axis shows the DV in standardized units, which corresponds to standardized *β* coefficients. The right *y*-axis shows the DV in the native units of each measure. Error bars reflect standard errors.

To do this, we ran two multiple regression models, one predicting % STEM Courses and the other predicting STEM Grades. The predictors of interest for each model were Math Anxiety and Math Ability. Within these models, we also included several predictors as covariates: Trait Anxiety, Verbal Working Memory, Gender, non-STEM Grades, and Semesters Absent. As an important additional covariate, each model also included the other STEM outcome—i.e., when % STEM Courses was the DV, we controlled for STEM Grades, and vice versa. Thus, the two models can be seen as tests for the extent to which the predictors of interest both uniquely *and independently* predict STEM participation and achievement. For full regression model details, see Table 1. The key results of these models are visualized in Fig. 1.

**Table 1:** Multiple regression models assessing the unique predictive effects of first-semester Math Anxiety and Math Ability on university STEM outcomes.

	(A) DV: % STEM Courses					(B) DV: STEM Grades				
Predictor	*B*	SE	*t*	*p*	*d*	*B*	SE	*t*	*p*	*d*
Math Anxiety	−.109	.026	−4.20	4E − 5	−.637	−2.406	.697	−3.45	7E − 4	−.524
Math Ability	.043	.023	1.91	.058	.290	.919	.602	1.53	.129	.232
% STEM Courses	–	–	–	–	–	-2.121	.652	-3.25	.001	−.493
STEM Grades	−.097	.030	−3.25	.001	−.493	–	–	–	–	–
Trait Anxiety	.026	.024	1.11	.267	.169	1.080	.623	1.74	.085	.263
Verbal Working Memory	−.004	.021	−.20	.841	−.031	.242	.542	.48	.655	.068
Gender	.002	.047	.03	.973	.005	.635	1.23	.52	.606	.078
non-STEM Grades	.157	.029	5.40	2E − 7	.819	7.579	.600	12.64	<2E − 16	1.92
Semesters Absent	.103	.021	4.98	2E − 6	.755	−.623	.585	−1.06	.289	−.161

Note: All predictors are standardized and the DV in each model is in its native units. The *B* estimates can therefore be interpreted as the change in actual % STEM Courses or STEM Grades associated with a one standard deviation increase in the predictor. *d* refers to Cohen’s *d* measure of effect size. For both models, *df* = 174. Table 1A adjusted *R*^2^ = .347. Table 1B adjusted *R*^2^ = .554.

The results displayed in Fig. 1a show that both Math Anxiety and Math Ability significantly predicted % STEM Courses when no variables were controlled for (zero-order effects). However, when controlling for one another and for relevant cognitive, affective, and academic measures, Math Anxiety robustly predicted unique variance in % STEM Courses (*β* = −.325, *t*(174) = −4.20, *p* = 4E − 5, Cohen’s *d* = −.637), but Math Ability did not (*β* = .129, *t*(174) = 1.91, *p* = .058, Cohen’s *d* = .290). These results indicate that even when holding Math Ability, STEM Grades, and all other covariates constant, a one standard deviation increase in Math Anxiety accounted for a 10.9% reduction in the proportion of STEM courses students chose to take. Given that students took an average of 36.13 courses over four years, this corresponds to a reduction of 3.93 STEM courses over four years of university, or about 1 course per year.

Similarly, the results displayed in Fig. 1b show that both Math Anxiety and Math Ability significantly predicted STEM Grades when no variables were controlled for (zero-order effects). When we tested whether these variables predicted unique variance in STEM Grades when controlling for each other and for other relevant covariates, we found that Math Anxiety predicted unique variance in STEM Grades (*β* = −.225, *t*(174) = −3.45, *p* = 7E − 4, Cohen’s *d* = −.524) and that Math Ability did not (*β* = .086, *t*(174) = 1.53, *p* = .129, Cohen’s *d* = .232). These results indicate that even holding Math Ability, % STEM Courses, non-STEM Grades, and all other covariates constant, a one standard deviation increase in Math Anxiety uniquely accounted for a 2.41 point reduction specifically in STEM Grades.

Together, the results of Fig. 1a and b show that, when controlling for relevant cognitive, affective, and academic variables, anxiety about math robustly predicted unique variance in both STEM outcomes of interest. To better contextualize these results, one can conceptualize individuals as ‘high’ and ‘low’ in Math Anxiety, corresponding to 1 standard deviation above and below the mean, respectively (note that this definition is in line with prior research^14,23^). From that perspective, our results indicate the average high math-anxious person would be expected to take an average of 7.86 fewer STEM courses (nearly 2 courses per year) and perform worse in those courses by 4.82 points (almost half of a letter grade) than their low math-anxious peers. Note this was true even after holding constant (i.e., controlling for) factors like objective math ability, general anxiety, verbal working memory, and relevant academic measures.

The results described thus far show that math anxiety measured in first-semester university students predicts independent variance in both STEM participation and achievement, over and above math ability and other important covariates. We next sought to further understand the predictive effects of math anxiety on these STEM outcomes by asking two additional questions of the data. First, we assessed the extent to which individual differences in math ability account for associations between math anxiety and STEM outcomes, and, conversely, the extent to which individual differences in math anxiety account for associations between math ability and STEM outcomes. Addressing this question can shed additional light on the extent to which relations between anxiety or ability in math are confounded by one another when predicting real-world STEM outcomes. Second, we sought to understand whether math anxiety was particularly predictive of STEM outcomes among particular types of students. This allowed us to address the question of for whom math anxiety may play an especially strong role in shaping university STEM outcomes.

### Secondary analysis 1: To what extent are associations between Math Anxiety and STEM outcomes accounted for Math Ability, and vice versa?

As discussed in detail above, a key limitation of much of the previous body of work establishing links between math anxiety and STEM participation and achievement is that, with few exceptions, individual differences in math ability are not controlled for, leaving these associations open to being confounded by math ability. However, this critique could also be applied in the opposite direction as well—there is a large body of work linking math ability to real-life STEM outcomes^15–20^, but because none of this work controls for differences in math anxiety, these findings are possibly confounded by math anxiety. Here, we sought to assess the extent to which the predictive effects of math anxiety on STEM outcomes could be accounted for by math ability and vice versa. To do so, we arranged our key math variables from the primary multiple regression results (Table 1, Fig. 1) in a mediation framework, which allowed us to directly quantify the extent to which math anxiety and math ability can account for one another’s association with each STEM outcome.

**Fig. 2:**
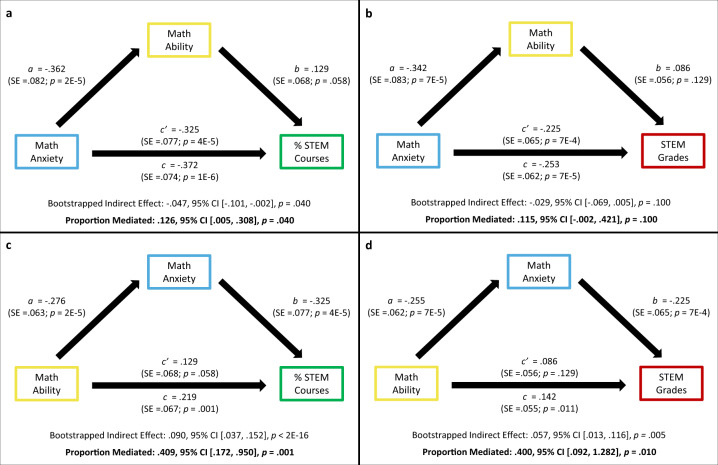
Mediation models showing the extent to which predictive effects of Math Anxiety on STEM outcomes can be accounted for by Math Ability, and vice versa. Figure 2 shows mediation models that assess the extent to which Math Anxiety and Math Ability can explain each other’s associations with % STEM Courses and STEM Grades. The following variables were controlled for in all models: Trait Anxiety, Verbal Working Memory, Gender, non-STEM Grades, and Semesters Absent. In models where % STEM Courses was the dependent variable (**a**, **c**), STEM Grades was included as an additional covariate. Likewise, in models where STEM Grades was the dependent variable (**b**, **d**), % STEM Courses was included as an additional covariate. ‘95% CI’ refers to bootstrapped 95% confidence intervals.

The mediation models were computed using the *mediation* package in R^25–27^. The strength and significance of all mediation models were tested using the bootstrapping method with 10,000 iterations^28^. Importantly, this analysis package provides an estimate of the *proportion mediated* (% C), which quantifies how much of the association between the independent variable (e.g., Math Anxiety) and the dependent variable (e.g., % STEM Courses) *can be attributed specifically to the presence of the mediator* (e.g., Math Ability) in the model. Proportion mediated (%C) is thus ideal for testing the extent to which relations between Math Anxiety and STEM outcomes are accounted for by Math Ability, and vice versa. The analyses are shown in Fig. 2a and b to test the extent to which *Math Ability* accounts for (i.e., reduces) the associations between Math Anxiety and % STEM Courses (Fig. 2a) and between Math Anxiety and STEM Grades (Fig. 2b). The analyses are shown in Fig. 2c and d test the opposite: the extent to which *Math Anxiety* accounts for (i.e., reduces) the associations between Math Ability and % STEM Courses (Fig. 2c) and between Math Ability and STEM Grades (Fig. 2d). Note that mediation models included the same covariates (Trait Anxiety, Verbal Working Memory, Gender, non-STEM Grades, and Semesters Absent) as the regression analyses in the previous section (see Table 1).

Mediation results in Fig. 2a and b show that only 12.6% of the association between Math Anxiety and % STEM Courses could be specifically attributed to Math Ability (%C = .126, CI_95_ of %C: .005, .308, *p* = .040); and only 11.5% of the association between Math Anxiety and STEM Grades was attributable to Math Ability (%C = .115, CI_95_ of %C: −.002, .421, *p* = .100). Note that only the former indirect effect reached traditional levels of significance (*p* = .040 and *p* = .100, respectively).

In contrast, the results from the mediation models displayed in Fig. 2c and d show that 40.9% of the association between Math Ability and % STEM Courses could be specifically attributed to Math Anxiety (%C = .409, CI_95_ of %C: .172, .950; *p* = .001); and 40.0% of the association between Math Ability and STEM Grades was attributable to Math Anxiety (%C = .400, CI_95_ of %C: .092, 1.282; *p* = .010). Both indirect effects were significant at traditional significance levels (*p* < 2E − 16, and *p* = .005, respectively).

To summarize, while there is some evidence to suggest that math ability partially accounts for associations between math anxiety and real-world STEM outcomes, the evidence supporting the reverse is stronger. Namely, these results suggest that math anxiety accounts for a substantial portion of the associations between math ability and real-world STEM outcomes.

### Secondary analysis 2: for whom is Math Anxiety most predictive of University STEM outcomes?

As a final set of analyses, we sought to whether specific types of students might be especially susceptible to the negative effects of math anxiety on STEM outcomes, as this could allow for better targeting of resources to prevent these possible negative outcomes. Specifically, we assessed whether the unique predictive effects of Math Anxiety on % STEM Courses and STEM Grades we observed in our primary analyses were moderated by two key variables of interest: non-STEM Grades and Gender. We used the marks students earned outside of STEM areas (i.e., non-STEM Grades) as a proxy for potential academic aptitude, reasoning that students who demonstrate strong academic aptitude in other areas may have untapped potential in STEM. This afforded the opportunity to test the idea that math anxiety is most pernicious in depressing STEM success among those who might otherwise be most likely to succeed. Namely, we tested whether those with higher non-STEM Grades would show a stronger unique relation between Math Anxiety and STEM outcomes. In addition, gender has been shown in past research to moderate the relation between math anxiety and *math* grades such that math anxiety is more predictive of math grades among men than women^5^. Here we tested whether this finding would generalize to *STEM* outcomes more broadly.

**Fig. 3:**
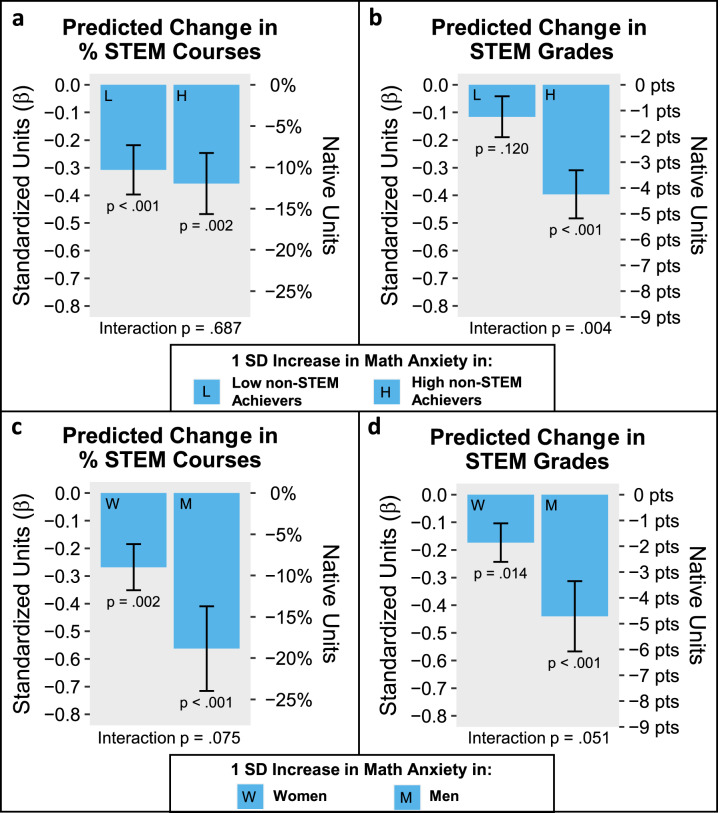
Testing moderators of the predictive effect of Math Anxiety on university STEM outcomes. Figure 3 shows the predicted change in STEM outcomes associated with a 1 SD (standard deviation) increase in Math Anxiety among different groups of students. The estimates are derived from multiple regression models that include all predictors (including Math Ability) used in Fig. 1, as well as the relevant interaction term. The *p*-value associated with the relevant interaction term (Fig. **a**–**b**: Math Anxiety × non-STEM Grades; Fig. 3**c**–**d**: Math Anxiety × Gender) is shown at the bottom of each figure. This interaction term in effect formally tests for a difference between the two bars in that subfigure. Figure **a** and **b** show the predicted change in STEM outcomes associated with a 1 SD increase in Math Anxiety in students with 1 SD below or above the mean in non-STEM academic achievement (high vs. low non-STEM Grades). Figure **c** shows the predicted change in STEM outcomes associated with a 1 SD increase in Math Anxiety in female and male students. In each figure, the left *y*-axis shows the DV in standardized units, which corresponds to standardized *β* coefficients. The right *y*-axis shows the DV in the native units of each measure. Error bars reflect standard errors.

All models that assessed moderation effects controlled for the same variables (Math Ability, Trait Anxiety, Verbal Working Memory, Gender, non-STEM Grades, Semesters Absent) that were controlled to assess the unique predictive effects shown in Fig. 1. We first asked whether the negative relation between Math Anxiety and STEM outcomes differed between students with relatively high or relatively low non-STEM achievement (measured by non-STEM Grades). Results showed that non-STEM Grades did not moderate the relation between Math Anxiety and % STEM Courses (*β* = −.025, *t*(173) = −.40, *p* = .687, Cohen’s *d* = −.061) but did moderate the relation between Math Anxiety and STEM Grades (*β* = −.140, *t*(173) = −2.90, *p* = .004, Cohen’s *d* = −.441). For full regression model details, see Tables S3 and S4. Separate effects for high and low non-STEM achievers are visualized in Fig. 3a (% STEM Courses) and Fig. 3b (STEM Grades). Figure 3b indicates that Math Anxiety is more negatively predictive of STEM Grades among students who demonstrate high aptitude in non-STEM courses relative to those with lower aptitude, possibly suggesting that higher levels of math anxiety may be holding students back from realizing their potential to succeed in STEM. These results indicate that among students with high non-STEM Grades, those high in math anxiety (1 standard deviation above the mean) would be predicted to score 8.48 points worse (or almost a full letter grade) than their low math-anxious (1 standard deviation below the mean) peers, even holding constant important factors like objective math ability, general anxiety, verbal working memory, and the number of STEM courses students choose to take.

We also asked whether Gender moderated the relation between Math Anxiety and either % STEM Courses or STEM Grades. From Fig. 3c–d, Math Anxiety tended to be more negatively predictive of university STEM outcomes among men than women, despite the fact that, consistent with the previous literature^21,29,30^, women reported significantly greater levels of Math Anxiety [*t*(181) = −5.35, *p* = 3E − 7, Cohen’s *d* = −.823]. That said, we urge strong caution in interpreting these results because the Gender × Math Anxiety term failed to reach traditional significance levels (*α* = .05) for either % STEM Courses (*β* = .295, *t*(173) = 1.79, *p* = .075, Cohen’s *d* = .272) or STEM Grades (*β* = .267, *t*(173) = 1.97, *p* = .051, Cohen’s *d* = .299). On the other hand, both effects were near the ‘significance’ threshold and had effect sizes (Cohen’s *d* in this case) of ~.28, which may be of interest to some readers. For full regression model details, see Tables S5 and S6.

## Discussion

Math anxiety is widely considered to be a barrier to success in STEM^1–3^. However, previous research supporting this idea has in fact provided only limited and indirect evidence that math anxiety is associated with STEM outcomes. By collecting math anxiety and math ability measures at the start of university, and by tracking objective and comprehensive STEM outcomes via four years of subsequent transcript data, we show that math anxiety collected in first-semester university students prospectively predicts both real-world university STEM participation and STEM achievement, even when controlling for individual differences in math ability. This work demonstrates clear support for the view that math anxiety plays a key role in suppressing STEM outcomes. This work also supports and further informs existing theory on the academically relevant consequences of math anxiety. The practical and theoretical implications of these findings, along with a discussion of their limitations, are described in detail below.

To our knowledge, the current results provide the most direct and robust evidence to date supporting links between math anxiety and two of its previously hypothesized consequences—avoidance of and underperformance in STEM. Zero-order associations showed robust associations between both math anxiety and math ability and both STEM outcomes (Fig. 1 zero-order effect). This result replicates previous work, while also highlighting the need to identify unique contributions of math anxiety and math ability to STEM outcomes. Importantly, we go beyond prior work by showing the relation between math anxiety and neither STEM avoidance nor STEM underperformance can simply be attributed to poor math skills (Fig. 1 unique effect). Furthermore, by focusing on a comprehensive list of all of the math-related courses students chose to take during their time at university as opposed to focusing more narrowly on a specific set of math courses, this work provides evidence that math anxiety is associated with both avoidance of and underperformance in STEM more broadly considered. In other words, this bolsters support for the idea that math anxiety may act as a barrier to *STEM* outcomes as opposed to simply *math* outcomes more narrowly.

Indeed, perhaps surprisingly, we found that math ability actually failed to predict unique variance in either STEM participation or achievement when individual differences in math anxiety were accounted for. These findings suggest that the way students feel about math, over and above their objective math ability, may be especially important for shaping decisions to avoid or pursue STEM topics, as well as for academic performance in those areas. As such, we suggest that future interventions developed with the aim of boosting STEM outcomes would do well to focus on math anxiety as a key leverage point.

Beyond their practical implications, these findings also inform theory on math anxiety in important ways. The finding that math anxiety predicted individual differences in STEM participation and achievement over and above math ability suggests that math anxiety is relevant for STEM outcomes independently of math anxiety’s already well-documented implications for math performance^4,5,12,13^. Indeed, when we assessed the extent to which associations between math anxiety and STEM outcomes could be explained by math ability, we found evidence that math ability accounted for only a relatively small portion of the associations between math anxiety and STEM outcomes (12.6% for STEM participation and 11.5% for STEM achievement; Fig. 2). Conversely, math anxiety accounted for 40.9% and 40.0% of the relations between math ability and STEM participation and STEM achievement, respectively. Taken together, these results suggest a need to update how we think about the specific ways and reasons why math anxiety may negatively affect STEM outcomes.

Avoidance of math is commonly assumed to be a consequence of math anxiety, but previous evidence supporting links between math anxiety and avoidance of math-related content has been limited. Much of the previous work that does exist relies on retrodictive associations between math anxiety and STEM avoidance, wherein math anxiety is measured after the avoidance behavior it is meant to explain. Moreover, most previous work has failed to control for differences in math ability, so it has been unclear whether it is truly anxiety toward math that is associated with avoidance of math, or whether it is simply ability in math that leads to avoidance of math-related areas. The present work addresses both of these limitations and shows that math anxiety predicts future avoidance of STEM over and above math ability. The scope of the present work is also broader than past work: we show that math anxiety predicts avoidance of STEM courses, broadly construed as courses that involve math, going beyond the more limited finding of previous work that math anxiety is associated with avoidance of math courses specifically^5^. These results thus provide some of the first clear evidence that highly math-anxious individual indeed avoids not just math courses, but STEM courses in general. Furthermore, this avoidance behavior cannot be attributed solely to poor math ability.

With respect to STEM achievement (grades), previous researchers have proposed two main ways in which math anxiety is thought to impact academic performance. In one account, math anxiety leads to avoidance of math over time, and this avoidance of math leads students to fail to fully develop their math skills, resulting in poor math ability^4^. This poor math ability then harms students’ ability to be successful in courses that require math. However, this account cannot explain the present findings—math anxiety predicted STEM Grades independently of differences in math ability, indicating that a more direct link between math anxiety and STEM achievement is needed.

Another possibility that operates in a more direct, real-time fashion is that students who are high in math anxiety experience an increase in state anxiety at the moment they have to do math-related tasks. This heightened anxiety then co-opts working memory resources that are necessary to complete difficult math tasks^1,4^, leading to a decrease in performance. For this explanation to be consistent with our observation that math ability did not mediate the relation between math anxiety and STEM achievement, it would need to be the case that the online effects of math anxiety (i.e., causing increased levels of state anxiety and worry when faced with math) are more pronounced during a true-stakes academic performance than during lab-based measures of math ability. Otherwise, the online effects of math anxiety on math performance would have already been captured in our lab-based measure of math ability, which, as we already noted, accounted for only a marginal portion of the relation between math anxiety and STEM Grades (Fig. 2). Thus, while our data cannot fully rule out this possibility, below we propose another possible explanation for our finding that math anxiety predicts STEM Grades independently of math ability, one that focuses on avoidance of math, but on a different scale than avoidance of math-related courses altogether.

While our results suggest that math-anxious students are more likely to avoid STEM courses when possible, importantly, we also showed that this avoidance of STEM courses cannot account for the observed robust relation between math anxiety and STEM Grades. This is because all models with STEM Grades as the outcome also included %STEM Courses as a covariate. Therefore, high-level decisions to avoid math-related courses—or what we term *macro-avoidance* of math—cannot explain poor STEM Grades. Instead, we propose that students with high levels of math anxiety may make shorter-term decisions to avoid math-related content—instances of what we call *micro-avoidance*. For instance, a highly math-anxious person might choose to focus less effort and attention on the more math-related elements of the STEM courses they take, leading to poorer grades in those courses. Importantly, this explanation is independent of students’ objective math ability, as well as their longer-term decisions to enroll in more or fewer STEM courses, thus making it consistent with the observed pattern of results demonstrating that math anxiety predicts STEM Grades even when controlling for math ability and the amount of STEM courses students chose to take. Instead, the crux of an explanation focusing on ‘micro-avoidances’ focuses more on how students choose to spend their limited time and effort in the courses in which they do enroll. This idea is of potentially broader theoretical consequence because research on math anxiety often discusses avoidance of math as a key consequence thereof, but researchers rarely provide greater specificity as to what this avoidance actually entails. In particular, high-level decisions to avoid math-related classes or careers and day-to-day (or even moment-to-moment) decisions to pay less attention in math class or expend less effort on math homework are often discussed interchangeably under the umbrella term “math avoidance”.

We should note that past work by Ashcraft and colleagues has introduced a distinction between types of math avoidance that is similar to the macro-avoidance vs. micro-avoidance distinction we make here. In Ashcraft and Faust^31^, for instance, the researchers find that math-anxious individuals tend to sacrifice accuracy in favor of speed on math tasks, which is interpreted as a form of “local avoidance of math” – this speed-accuracy tradeoff presumably reflects math-anxious individuals deciding not to expend effort on the math task they are asked to complete, instead of wishing to get through it as soon as possible. The idea of local avoidance of math (speed-accuracy tradeoffs while engaging in math tasks) as distinguished from global avoidance of math (avoiding math courses and career paths altogether) is raised in some of Ashcraft and colleagues’ later work as well^4,31,32^. On the one hand, we believe the terms ‘macro-avoidance’, as described here, and ‘global avoidance’, as described by Ashcraft and colleagues, are largely synonymous. On the other hand, while “local avoidance” of math has thus far in the literature referred specifically to the idea of speed-accuracy tradeoffs during math tasks, here we propose the idea of “micro-avoidance” of math that would also include relevant academic behaviors like paying less attention in class, skipping class altogether, studying less, etc. Returning to our interpretation of the relation between math anxiety and STEM achievement (controlling for math ability), if math-anxious individuals are likely to engage in class-related micro-avoidance behaviors in courses that involve math, this would make them less likely to be successful in these courses.

To unpack this hypothesis in greater detail, most of the work linking math anxiety to avoidance of math (including the present work) has focused on high-level avoidance behaviors (macro-avoidance)—avoidance of classes, majors, and careers that involve math^5–9^. This can be contrasted with the micro-avoidance of math, which refers not to high-level decisions about whether to pursue educational paths or careers that involve math, but instead to small-scale decisions about how much attention to pay in math class, how much effort to exert on math homework, and so on. Micro-avoidance behaviors like these are often assumed to lead math-anxious elementary-aged children (who, importantly, are all enrolled in math courses) to fail to fully develop their math abilities by causing students to get less practice (or less effective practice) with math over time^1,4,33,34^. However, only recently have studied directly assessed whether math anxiety is associated with micro-avoidance of math at all. Work by Pizzie and Kraemer^35^ showed that math anxious individuals tended to shift their attention away from complicated mathematical formulas even when they were not required to do any math. A recent set of studies by Choe, Jenifer et al.^36^ showed that math-anxious individuals have a tendency to avoid doing difficult math even when doing so entails a monetary cost. Together these studies suggest that math anxiety may indeed be associated with micro-avoidance of math, but on their own, they do not provide evidence that these micro-avoidance behaviors would occur in actual educational contexts. One recent study has begun to fill that gap by directly asking whether math anxiety does, in fact, predict the amount of attention and effort students expend in math-related courses^37^. In that study, researchers found that among seventh-grade students, math anxiety predicted the extent to which students paid attention in math class, which in turn predicted the development of their math abilities. Speculatively applying this finding to the present study, it may be the case that math anxiety led students enrolled in STEM courses to engage with or attend sub-optimally to math-related content in those courses, which in turn could explain variability in grades in those courses, over and above both math ability and macro-level avoidance of STEM content. In general, we see further exploration of micro-avoidance of math as a fruitful avenue for future research that attempts to understand the possible mechanisms by which math anxiety affects academic achievement.

More broadly, when considering why math anxiety would predict STEM achievement over and above math ability, we believe it is useful to consider the following question—what determines how successful a student will be in a STEM course? There is sometimes a tendency to assume that performance in a course is determined simply by how “good” a student is at the course material, which places the focus squarely on ability. However, there are clearly many factors that would determine how well a student does in a course: how often they attend class, how often they do the reading, how often they study, how much attention they devote to each of these things while they are doing them. These types of behaviors represent what Eccles, Wigfield, and colleagues refer to as “achievement-related choices”^38–41^. There are many more determinants of performance within a specific class, of course, but the key point here is that what determines an individual’s final grade in a course is perhaps determined as much by how much effort they put into the course as their preexisting abilities in the relevant domain. We suggest here that math anxiety may predict STEM achievement over and above math ability because math anxiety is more likely to be directly associated with a tendency to avoid expending effort in courses that involve math.

It should be noted that the approach taken in this paper is different from past work assessing the extent to which math anxiety and math grades in one year of schooling predict math anxiety and math grades in future years (e.g., Meece, Wigfield, and Eccles^6^) in that the goal was not to assess how past *grades* in math-related courses predict achievement and participation levels in future math-related courses alongside math anxiety, but rather how a measure of math *ability* predicts these important outcomes alongside math anxiety. Previous work has shown that past grades tend to be strong predictors of future grades^6,38–40^. However, it is important to interrogate *why* past academic performance would predict future academic performance—how much of it is because students have a set ability level for that kind of course, and how much of it is a result of things like how much effort a student chooses to expend in that course? By collecting a measure of math ability at the beginning of university alongside our measure of math anxiety, here we were able to isolate preexisting differences in math ability as a possible explanation of differences in future STEM performance. It is of course possible that math-anxious students’ past struggles in math-related courses can in part explain why they are math-anxious in the first place (for a review, see Ramirez, Shaw, and Maloney^42^). Importantly, however, the present results indicate that math anxiety was a better predictor of future STEM achievement than math ability, suggesting that while math-anxious students do, on average, have lower math ability than their less-anxious counterparts, these preexisting differences in math ability cannot explain why math-anxious students ultimately do worse in STEM courses. This indicates that other mechanisms are at play, and we hypothesize that the kinds of class-relevant micro-avoidance behaviors described here are one such mechanism.

In addition to assessing whether math *ability* can explain associations between math *anxiety* and STEM outcomes, we also examined the opposite: whether math *anxiety* could explain associations between math *ability* and STEM outcomes. Math ability is often discussed as a possible confound for associations between math anxiety and real-world outcomes, but the opposite—that math anxiety may actually confound relations between math ability and real-world outcomes—is seldom mentioned but in principle no less likely. Our results showed that math anxiety accounted for approximately 40% of the associations between math ability and both STEM participation and achievement (or about 3–4 times what math ability was able to account for of the associations between math anxiety and STEM outcomes). These findings suggest that, while math ability does predict differences in these important university STEM outcomes, a large portion of those associations can be explained by math anxiety. On a practical level, this suggests that interventions hoping to boost these STEM outcomes perhaps ignore students’ *anxiety* about math at their peril.

More broadly, this finding has significant implications for previous work linking differences in math ability to real-world outcomes like grades earned in math courses^15^, attainment of STEM careers^16–18^, and income levels and health outcomes^19,20^. Just as we have pointed out that a great deal of previous work linking math anxiety to real-world outcomes did not control for differences in math ability, the vast majority of work linking Math Ability to these important real-world outcomes did not control for differences in math anxiety. Although a subset of these studies did control for other math attitudes, like math confidence^19^, none of this previous work, examined the extent to which associations between math ability and real-world outcomes could be *accounted for* by attitudes—positive or negative—toward math. Our present findings suggest the real possibility that many previously observed links between math ability and real-world outcomes may be, at least in part, explainable by negative attitudes toward math—namely, math anxiety. Thus, we propose future research revisit some of these earlier findings to understand the extent to which it is truly ability in math, rather than feelings of anxiety toward math, that better account for these various real-world outcomes. Doing so would not only inform theory on the ways in which math ability and math anxiety shape future outcomes, but it would also allow for better-targeted interventions to focus more on math ability or anxiety depending on what the evidence points to.

The present work also demonstrated that math anxiety *independently* predicted two key academically relevant consequences—avoidance of and underperformance in math-related courses. This was evidenced by the finding that math anxiety continued to negatively predict the proportion of STEM courses students took even controlling for STEM Grades, and vice versa. The predictive effects of math anxiety on avoidance of STEM can therefore not be explained by recent underperformance in STEM, and vice versa. This suggests that both of math anxiety’s key theorized academic consequences—avoidance of and underperformance in math-related coursework—operate independently to shape academic outcomes, suggesting the need to posit separate potential mechanisms to account for these disparate effects (as we have done in the preceding sections). From an intervention standpoint, it may therefore be the case that an intervention that reduces the impact of math anxiety on one outcome (underperformance in STEM, for instance) would be unlikely to reduce the impact of math anxiety on the other outcome (avoidance of STEM). Several recent math anxiety interventions have focused not on reducing Math Anxiety levels directly, but on alleviating math anxiety’s negative effects on math performance. The expressive writing intervention, for instance, in which students write about their worries about an upcoming math test just before the test begins, aims to help students deal with math anxiety-induced worry before the test, and the goal is to boost test scores^43^. The same is true for cognitive reappraisal-based interventions, in which participants are instructed to think about the situation differently in an effort to reduce in-the-moment anxiety levels^44^. Even if these interventions were scaled up and found to be effective at boosting STEM Grades, our finding that math anxiety predicts avoidance of STEM courses over and above STEM Grades suggests that these interventions would be unlikely to affect student decisions to enroll in more STEM courses. The present results suggest that to effectively intervene to boost both STEM participation and STEM achievement of math-anxious individuals, interventions would need to be developed that either focuses separately on both of these outcomes or that attempt to reduce math anxiety levels directly.

We also found evidence that the negative association between math anxiety and STEM Grades was strongest among students with high academic performance in non-STEM courses (Fig. 3b). This result supports the notion that math anxiety can prevent otherwise high-achieving students from realizing their potential in STEM, suggesting that math anxiety may be a particularly pernicious contributor to the ‘leaky pipeline’ in STEM, possibly preventing talented students from succeeding in STEM courses. One possible explanation for this finding may be that for otherwise high-achieving students, the online negative effects of math anxiety on performance may be especially pronounced. This would be consistent with work showing that high-aptitude students are often the ones who are most harmed by anxiety and pressure^22^. Another possible explanation for this finding relates back to the possibility that math anxiety predicts STEM Grades largely by determining how students choose to spend their limited time and resources. It is possible, for instance, that students who have a high non-STEM aptitude and are high in math anxiety may be especially likely to choose to devote more of their limited time and effort to non-STEM and devote less of their attention to the courses they are enrolled in that involve math.

While this study addresses several important limitations of previous work linking math anxiety to academic STEM outcomes, its own limitations should also be noted. The present study takes care to rule out confounds by controlling for several covariates (including math ability, general Trait Anxiety, working memory, and non-STEM Grades), and it also addresses previous issues of directionality by collecting our measure of math anxiety at the beginning of students’ time at university before decisions to pursue or avoid STEM and performance levels in STEM courses had occurred (making it impossible that individual differences in university-level STEM outcomes caused differences in math anxiety). However, the design is still fundamentally correlational, limiting our ability to make strong inferences about the causal effects of math anxiety on STEM outcomes. We see this work as strongly suggesting that math anxiety may play a causal role in determining STEM outcomes, but intervention-based work that aims to reduce the effect of math anxiety on these STEM outcomes is needed to conclusively infer that Math Anxiety can cause the specific STEM outcomes we considered here. In addition, this study focused on students at a large public Canadian university, and future work would need to be done to assess whether the findings would generalize to different educational contexts.

Another limitation of this work is that our measure of math ability—a difficult mental arithmetic task—does not capture all of the possible types of math skills (reasoning about mathematics, for instance) that may affect success in STEM courses. As we note in the Methods section, we chose this as our math ability measure because all students were likely to have the requisite knowledge to complete the task (i.e., it does not rely on knowledge of advanced math, like calculus) and because this type of foundational math skill is likely to be broadly applicable across almost all types of specialized math that students are likely to encounter in any STEM discipline. Moreover, past work has demonstrated that mental arithmetic ability is a reliable predictor of ability in more complex math^45,46^. Nevertheless, it remains possible that ability on other types of math measures may explain, in part, the association between math anxiety and STEM outcomes. However, given the association between ability in arithmetic and more complex math skills, there would need to be something specific about these more advanced math skills that explain the association between math anxiety and STEM outcomes that are not captured by ability on the complex mental arithmetic task we used here. Future work could be done to explore whether other forms of math ability could explain the predictive associations between math anxiety and STEM outcomes, and such work would refine our theoretical understanding of the association between math anxiety and STEM outcomes. The present work, however, provides an important demonstration that the predictive effects of math anxiety on university STEM outcomes are largely independent of its effects on difficult mental arithmetic.

Together, the present findings provide some of the strongest evidence to date that math anxiety, over and above math ability, serves as a barrier to multiple facets of STEM success, supporting key predictions made by theory on math anxiety. Specifically, we show that predictive effects on STEM achievement are not explainable by differences in math ability, prompting a reevaluation of the mechanisms by which math anxiety may lead to poor performance in math-related courses. We also provide evidence that math anxiety can in fact account for associations between math ability and real-world outcomes, suggesting that math anxiety may confound previously observed associations between math ability and real-world outcomes that largely ignored math anxiety. Moreover, we show that math anxiety predicts STEM achievement and participation independently of one another, suggesting that the effects of math anxiety on avoidance of and underperformance in STEM can operate through separate mechanisms. Finally, we found evidence that math anxiety is particularly negatively predictive of STEM achievement for those who have high non-STEM Grades. In sum, while this correlational study cannot establish a causal role for math anxiety shaping university-level STEM participation and achievement, it provides clear theoretical grounds for devoting resources to test whether interventions that alleviate the negative effects of math anxiety as students enter university can bolster their STEM outcomes.

## Methods

### Participants

One hundred and eighty-six first-year undergraduates at the University of Western Ontario participated. Participants were recruited widely, both through flyers posted throughout campus and through research assistants recruiting students directly at places where many students congregate. All first-year students were eligible to participate. We recruited as many participants as possible within students’ first semester of university—this limitation was made to enable us to assess the extent to which math anxiety and other variables collected at the start of university prospectively predicted student outcomes throughout their time at university. Of this initial sample, three were removed from all analyses either because they were not actually a first-year student or because they missed more than one-third of attention checks, resulting in a total analytic sample size of 183 (117 female; mean age = 18.55, SD = .41). Power analysis (assuming power of .80 and an alpha level of .05) showed that this sample was suitable to detect correlation effect sizes as small as Pearson’s *r* values of .205. Moreover, power analysis for multiple regression effects showed that a sample size of 183 with 9 predictors (the most complex analysis we ran) is powered to find Cohen’s *f*^2^ effect sizes as small as .07, which is considered a small effect size^47^ (Cohen, 1988). The sample size was thus deemed appropriate to detect practically significant effects.

It should be noted that the data reported here are part of a larger dataset, some of which have been reported on in previous work^21,48^. The theoretical questions addressed and the analyses described in this manuscript are novel.

### Procedure

All participants provided written consent, and the University of Western Ontario Ethics Review Board approved all data collection procedures. In their first semester of university, participants completed a battery of questionnaires and cognitive tasks in the lab. Participants have compensated $20 CAD for their time. The order of surveys and cognitive tasks was counterbalanced and randomized across participants. In addition to completing this two-hour lab session, participants also granted the researchers permission to access their de-identified academic transcripts throughout their undergraduate careers by indicating as such on the consent form. These transcripts listed the courses students chose to take and the grades they earned in those courses.

### Materials

#### % STEM Courses

The academic transcripts listed all courses students completed throughout their four years at university and the department in which that course was offered. Because the goal of this work is to understand the extent to which math anxiety predicts STEM outcomes, we first categorized different departments as STEM or non-STEM. This was done in two steps. First, traditional science and math departments (e.g., Physics, Mathematics, Engineering, Chemistry) were classified as belonging to the STEM category. Second, for more ambiguous departments (e.g., those in the social sciences, such as Psychology, Economics, Sociology, etc.), the STEM/non-STEM classification was made based on an examination of the content of courses within that department in consultation with university students directly familiar with those courses. The main consideration taken here was whether the courses offered in a given department as a whole involved a substantial amount of math content and/or content and materials about the application of the scientific method. As an illustrative example of these more ambiguous cases, take the two departments Business Administration and Financial Modeling. The content of courses in both departments has to do with theory and practice in business, but Financial Modeling was designated as STEM because of the heavy computational components of many of the courses in that department while Business Administration was designated as non-STEM because its courses did not in general place a large focus on mathematics. For a full list of the departments that participating students took courses in and their STEM/non-STEM designation, see Table S1. Note that while this process inherently involves a degree of subjectivity, departments were designated as STEM or non-STEM well before we conducted any analyses using this transcript data, and no changes to the STEM/non-STEM designations were made after analyses began.

The ‘% STEM Courses’ measure was computed by summing the number of STEM courses taken by each participant and dividing it by the total number of courses that students took. A proportion was used instead of the raw number of STEM courses to account for variation in the total number of courses, and thus control for the number of non-STEM courses taken by each student. It is useful to note that this latter point is why regression models do not include a control variable ‘% Non-STEM courses’, as % Non-STEM would simply be 1–%STEM.

#### STEM Grades

For each course taken, transcripts listed the grade the student earned in the course from 0 to 100 points. For each participant, a STEM Grades score was created by computing the average of scores earned in STEM courses.

In some cases, transcript data indicated that students repeated a course they had taken previously, either because they withdrew from the course the first time they took it or because they obtained a poor grade the first time they took it and needed to retake the course to fulfill degree requirements. Of the total of 6211 courses taken by all students in our sample, 52 of those courses (0.8%) were repeated courses, and a total of 34 out of 183 students (18.6%) repeated a course at least once. Controlling for the number of repeated courses students took does not substantially affect any of the key estimates or inferences presented. Hence, to maximize the use of the extant data, we included all grades recorded for a given student on their transcript, regardless of repeats (i.e., when computing the STEM Grades and Non-STEM Grade measures).

#### Math Anxiety

Math Anxiety was measured using the short Math Anxiety Rating Scale (sMARS^49^). Participants rated how anxious they would feel in 25 math-related situations (e.g., “Studying for a math test”; “Being given a set of division problems to solve on paper”), which was scored from 0 (Not at all) to 4 (Very much). Math Anxiety scores range from 0 to 100. Cronbach’s α for this measure was .96.

#### Math Ability

Participants completed difficult mental arithmetic problems adapted from the Kit of Factor-Referenced Cognitive Tests^50,51^. Trials included all four basic arithmetic operations: addition (three 2-digit numbers; e.g., 45 + 72 + 87), subtraction (a 2-digit or 3-digit minuend and a 2-digit or 3-digit subtrahend; e.g., 354–87), multiplication (one 2-digit number and one 1-digit number; e.g., 64 × 6), and division (a 1-digit divisor into a 2-digit or 3-digit dividend; e.g., 432 ÷ 9). Problems were open-ended (i.e., not verification); hence, participants responded by typing their answers using the number pad on the keyboard. They were required to calculate the answer mentally—that is, pencil and paper or other devices were not permitted to aid with calculation. As such, the task was relatively difficult for arithmetic (mean accuracy = 81.2%, mean RT = 9.91 s). Operation types were presented in separate blocks, and in each block, participants completed as many problems as they could in 3 min. Participants were not aware that there was a time limit, and the block ended once a participant completed the trial they were at once 3 min had passed (this final trial was omitted from analysis). A math ability score was computed for each participant by summing the total number of problems answered correctly across all four operation types, where higher scores indicate greater math ability. Past work has shown that performance on this task is correlated with performance on several basic numerical tasks (including numerical ordering and numerical comparison tasks^51^). Internal reliability for this task was computed using participants’ scores for each of the four operation types; Cronbach’s α was .89.

While we acknowledge that mathematics as a whole comprises far more than even the most difficult mental arithmetic task, here we chose a challenging arithmetic task as a measure of objective math ability for several reasons. First, this ensured that all participants would have the requisite knowledge to complete the chosen task (e.g., it is not a given that all students would have taken the necessary course for more specialized math topics, such as geometry or calculus). Second, not all STEM courses will require the same type of specialized math or even specialized math at all. That is, arithmetic is likely to be one of the more universally tapped math skills across a wide range of STEM courses. Finally, prior empirical work has shown that arithmetic is a reliable predictor of more advanced math skills^45,46^.

#### Trait Anxiety

Trait Anxiety was measured using the Trait Anxiety Inventory (TAI^52^). Participants respond to statements like “I worry too much over something that doesn’t really matter” and “I am ‘calm, cool, and collected’” (reverse scored) on a scale from 1 (Almost never) to 4 (Almost always) based on how they generally feel. The scale contains a total of 20 items, and possible scores range from 20 to 80, where higher scores indicate greater general anxiety. Trait Anxiety was included as a covariate to control for anxiety that is not specific to math. Cronbach’s α for this measure was .93.

#### Verbal Working Memory

Verbal Working Memory capacity was measured using the commonly used automated reading-span task^53,54^. In this task, participants saw a series of sentences and had to judge whether each sentence is sensible (e.g., “Andy was stopped by the policeman because he crossed yellow heaven”). After each sentence, a letter appeared on the screen. After a sequence of 3 to 7 sentences followed by letters, participants recalled the letters they saw in the correct order by clicking them on a new screen that displayed a menu of letters. Participants completed a total of 15 sequences, 3 of each possible sequence length in a randomized order. For each sequence, a participant’s score was determined by multiplying accuracy by length, where accuracy is a binary indicator (1 or 0) of whether all letters were recalled in the correct order, and length is the number of letters participants had to recall in that sequence. A total Verbal Working Memory score was calculated for each participant by summing the scores from each trial. Verbal Working Memory scores range from 0 to 75, where higher scores reflect higher Verbal Working Memory capacity. The Verbal Working Memory task was included as a covariate to control for general cognitive ability.

#### Gender

To control for gender, participants responded to an item asking them to indicate their gender (Male or Female; coded as 0 or 1, respectively).

#### Non-STEM Grades

For each participant, a non-STEM Grades score was created by computing the average of scores (from 0 to 100 points) earned in non-STEM courses. This allowed us to control for performance in non-STEM courses.

#### Semesters Absent

At least one semester of transcript data was missing for a subset of 35 participants in the final analytic sample, either because the student took time off from school or left the university. To enable us to account for this in our analysis, we created a ‘Semesters Absent’ control variable that indicates how many semesters of transcript data were missing for a given participant. Note that the results presented in this work do not appreciably change if participants who are missing any transcript data are simply excluded from analyses.

### Reporting summary

Further information on research design is available in the Nature Research Reporting Summary linked to this article.

## Supplementary information

Supplementary Information

Reporting Summary

## Data Availability

The data supporting this work can be found at the following link: https://osf.io/bctyg/?view_only=9ce7dd98a8464f1db2af1a40eb0336e7/.
