# Supplementary material for: First-year students’ math anxiety predicts STEM avoidance and underperformance throughout university, independently of math ability
Source: NPJ Sci Learn. 2021 Jun 14;6:17. doi: 10.1038/s41539-021-00095-7 (PMC8203776; doi:10.1038/s41539-021-00095-7)
Supplement: Supplementary file 1 — Supplementary Information [file 41539_2021_95_MOESM1_ESM.pdf]

Supplementary Information

Supplementary Table 1

Department Name	STEM
Actuarial Science	1
American Studies	0
Anthropology	0
Applied Mathematics	1
Arts and Humanities	0
Astronomy	1
Biology	1
Business Administration	0
Calculus	1
Centre for Global Studies	0
Chemistry	1
Chinese	0
Classical Studies	0
Comparative Literature and Culture	0
Computer Science	1
Digital Humanities	0
Dimensions of Leadership	0
Earth Sciences	1
Economics	0
English	0
Engineering Science	1
Environmental Science	1
Family Studies and Human Development	0
Film Studies	0
Financial Modeling	1
Foods and Nutrition	0
French	0
Geography	0
German	0
Health Sciences	0

Department Name	STEM
History	0
Interdisciplinary Studies	0
Italian	0
Japanese	0
Kinesiology	0
Mathematics	1
Medieval Studies	0
Media, Information and Technoculture	0
Management and Organizational Studies	0
Music	0
Philosophy	0
Physics	1
Physiology	1
Political Science	0
Portuguese	0
Psychology	0
Religious Studies	0
Russian	0
Scholars Electives	0
Sociology	0
Social Justices and Peace Studies	0
Social Work	0
Spanish	0
Speech	0
Statistical Sciences	1
Visual Arts History	0
Visual Arts Studio	0
Women's Studies	0
Writing	0

Note: The list of academic departments in which participating students took courses. Each department was classified as either STEM (1) or non-STEM (0). For details on this classification, see the Methods section.

Supplementary Table 2

Measure	Mean (SE)	% STEM Courses	STEM Grades	Math Anxiety	Math Ability	Trait Anxiety	Verbal Working Memory	Gender	non-STEM Grades
% STEM Courses	.48 (.02)	-							
STEM Grades	77.81 (.79)	.12	-						
Math Anxiety	30.62 (1.52)	-.39***	-.32***	-					
Math Ability	51.1 (1.82)	.24***	.28***	-.35***	-				
Trait Anxiety	40.84 (.79)	-.13	-.10	.43***	.03	-			
Verbal Working Memory	46.27 (1.16)	.01	.06	-.07	.17*	-.07	-		
Gender	.64 (.04)	-.08	-.04	.37***	-.13	.32***	-.09	-	
non-STEM Grades	79.16 (.66)	.34***	.71***	-.29***	.22**	-.20**	.02	-.02	-
Semesters Absent	.51 (.08)	.33***	-.18*	-.05	-.08	-.02	-.05	.08	-.08

Note: Descriptive statistics and zero-order correlations for all variables are shown. $N = 183$. * $p \leq .05$, ** $p \leq .01$, *** $p \leq .001$

Supplementary Table 3

DV: % STEM Courses	B	SE	*t*	*p*	*d*
Math Anxiety	-.111	.027	-4.17	5E-5	-.635
Non-STEM Grades	.044	.023	1.93	.055	.293
Math Ability	-.100	.031	-3.26	.001	-.495
STEM Grades	.028	.024	1.17	.245	.177
Trait Anxiety	-.005	.021	-.24	.807	-.037
Verbal Working Memory	.001	.047	.02	.988	.002
Gender	.160	.030	5.31	3E-7	.808
Semesters Absent	.103	.021	4.95	2E-6	.753
Math Anxiety x non-STEM Grades	-.008	.021	-.40	.687	-.061

Note: All predictors are standardized and % STEM Courses is in its native units. The B estimate can therefore be interpreted as the change in % STEM Courses associated with a one standard deviation of the predictor. *d* refers to Cohen's *d* measure of effect size. $df = 173$. Adjusted $R^2 = .343$.

Supplementary Table 4

DV: STEM Grades	B	SE	*t*	*p*	*d*
Math Anxiety	-2.743	.692	-3.96	1E-4	-.603
Non-STEM Grades	.984	.590	1.67	.097	.253
Math Ability	-2.080	.639	-3.26	.001	-.495
% STEM Courses	1.374	.618	2.22	.028	.338
Trait Anxiety	.064	.534	.12	.905	.018
Verbal Working Memory	.440	1.207	.36	.716	.055
Gender	7.817	.593	13.19	< 2E-16	2.00
Semesters Absent	-.630	.573	-1.10	.273	-.167
Math Anxiety x non-STEM Grades	-1.503	.519	-2.90	.004	-.441

Note: All predictors are standardized and STEM Grades is in its native units. The B estimate can therefore be interpreted as the change in STEM Grades associated with a one standard deviation of the predictor. *d* refers to Cohen's *d* measure of effect size. $df = 173$. Adjusted $R^2 = .571$.

Supplementary Table 5

DV: % STEM Courses	B	SE	*t*	*p*	*d*
Math Anxiety	-.188	.051	-3.67	3E-4	-.558
Math Ability	.046	.023	2.02	.045	.307
STEM Grades	-.103	.030	-3.46	.001	-.527
Trait Anxiety	.031	.024	1.31	.192	.199
Verbal Working Memory	-.011	.021	-.55	.582	-.084
Gender	.030	.049	.62	.535	.095
non-STEM Grades	.162	.029	5.58	9E-8	.849
Semesters Absent	.108	.021	5.19	6E-7	.789
Math Anxiety x Gender	.099	.055	1.79	.075	.272

Note: All predictors are standardized and % STEM Courses is in its native units. The B estimate can therefore be interpreted as the change in % STEM Courses associated with a one standard deviation of the predictor. *d* refers to Cohen's *d* measure of effect size. $df = 173$. Adjusted $R^2 = .355$.

Supplementary Table 6

DV: STEM Grades	B	SE	*t*	*p*	*d*
Math Anxiety	-4.71	1.361	-3.46	6E-4	-.527
Math Ability	.988	.598	1.65	.100	.251
% STEM Courses	-2.25	.650	-3.46	.001	-.527
Trait Anxiety	1.206	.621	1.94	.054	.295
Verbal Working Memory	.023	.549	.04	.967	.006
Gender	1.457	1.290	1.13	.260	.172
non-STEM Grades	7.640	.595	12.83	< 2E-16	1.951
Semesters Absent	-.430	.589	-.73	.466	-.111
Math Anxiety x Gender	2.856	1.452	1.97	.051	.299

Note: All predictors are standardized and STEM Grades is in its native units. The B estimate can therefore be interpreted as the change in STEM Grades associated with a one standard deviation of the predictor. *d* refers to Cohen's *d* measure of effect size. $df = 173$. Adjusted $R^2 = .561$.
