# Supplementary material for: First-year students’ math anxiety predicts STEM avoidance and underperformance throughout university, independently of math ability
Source: NPJ Sci Learn. 2021 Jun 14;6:17. doi: 10.1038/s41539-021-00095-7 (PMC8203776; doi:10.1038/s41539-021-00095-7)
Supplement: Supplementary file 2 — Reporting Summary [file 41539_2021_95_MOESM2_ESM.pdf]

Corresponding author(s): Richard Daker
Last updated by author(s): Mar 22, 2021

Reporting Summary

Nature Research wishes to improve the reproducibility of the work that we publish. This form provides structure for consistency and transparency in reporting. For further information on Nature Research policies, see our Editorial Policies and the Editorial Policy Checklist.

Statistics

For all statistical analyses, confirm that the following items are present in the figure legend, table legend, main text, or Methods section.

n/a	Confirmed	
☐	☒	The exact sample size (*n*) for each experimental group/condition, given as a discrete number and unit of measurement
☐	☒	A statement on whether measurements were taken from distinct samples or whether the same sample was measured repeatedly
☐	☒	The statistical test(s) used AND whether they are one- or two-sided<br>*Only common tests should be described solely by name; describe more complex techniques in the Methods section.*
☐	☒	A description of all covariates tested
☐	☒	A description of any assumptions or corrections, such as tests of normality and adjustment for multiple comparisons
☐	☒	A full description of the statistical parameters including central tendency (e.g. means) or other basic estimates (e.g. regression coefficient) AND variation (e.g. standard deviation) or associated estimates of uncertainty (e.g. confidence intervals)
☐	☒	For null hypothesis testing, the test statistic (e.g. *F*, *t*, *r*) with confidence intervals, effect sizes, degrees of freedom and *P* value noted<br>*Give P values as exact values whenever suitable.*
☒	☐	For Bayesian analysis, information on the choice of priors and Markov chain Monte Carlo settings
☒	☐	For hierarchical and complex designs, identification of the appropriate level for tests and full reporting of outcomes
☐	☒	Estimates of effect sizes (e.g. Cohen's *d*, Pearson's *r*), indicating how they were calculated

Our web collection on statistics for biologists contains articles on many of the points above.

Software and code

Policy information about availability of computer code

Data collection	EPrime Version 2 was used for all cognitive measures. Qualtrics was used for survey measures.
Data analysis	R version 4.0.2

For manuscripts utilizing custom algorithms or software that are central to the research but not yet described in published literature, software must be made available to editors and reviewers. We strongly encourage code deposition in a community repository (e.g. GitHub). See the Nature Research guidelines for submitting code & software for further information.

Data

Policy information about availability of data

All manuscripts must include a data availability statement. This statement should provide the following information, where applicable:

- Accession codes, unique identifiers, or web links for publicly available datasets
- A list of figures that have associated raw data
- A description of any restrictions on data availability

The data supporting this work can be found at the following link: https://osf.io/bctyg/?view_only=9ce7dd98a8464f1db2af1a40eb0336e7\

Field-specific reporting

Please select the one below that is the best fit for your research. If you are not sure, read the appropriate sections before making your selection.

☐ Life sciences ☒ Behavioural & social sciences ☐ Ecological, evolutionary & environmental sciences

For a reference copy of the document with all sections, see nature.com/documents/nr-reporting-summary-flat.pdf

Behavioural & social sciences study design

All studies must disclose on these points even when the disclosure is negative.

Study description	All data are quantitative
Research sample	One hundred and eighty-six first-year undergraduates at the University of Western Ontario participated. Participants were recruited widely, both through flyers posted throughout campus and through research assistants recruiting students directly at places where many students congregate. All first-year students were eligible to participate. We recruited as many participants as possible within students' first semester of university – this limitation was made to enable us to assess the extent to which math anxiety and other variables collected at the start of university prospectively predicted student outcomes throughout their time at university.
Sampling strategy	This was a convenience sample. Of this initial sample, three were removed from all analyses either because they were not actually a first-year student or because they missed more than one third of attention checks, resulting in a total analytic sample size of 183 (117 female; mean age = 18.55, SD = .41). Power analysis (assuming power of .80 and an alpha level of .05) showed that this sample was suitable to detect correlation effect sizes as small as Pearson's r values of .205. Moreover, power analysis for multiple regression effects showed that a sample size of 183 with 9 predictors (the most complex analysis we ran) is powered to find Cohen's f2 effect sizes as small as .07, which is considered a small effect size (Cohen, 1988). The sample size was thus deemed appropriate to detect practically significant effects.
Data collection	All data collection took place on the computer. Only the researcher and participant were in the room. There were no experimental conditions.
Timing	All behavioral and survey data was collected in the Fall of 2014.
Data exclusions	Of this initial sample, three were removed from all analyses either because they were not actually a first-year student or because they missed more than one third of attention checks, resulting in a total analytic sample size of 183 (117 female; mean age = 18.55, SD = .41).
Non-participation	No participants dropped out.
Randomization	There were no experimental groups.

Reporting for specific materials, systems and methods

We require information from authors about some types of materials, experimental systems and methods used in many studies. Here, indicate whether each material, system or method listed is relevant to your study. If you are not sure if a list item applies to your research, read the appropriate section before selecting a response.

Materials & experimental systems

n/a	Involved in the study	
☒	☐	Antibodies
☒	☐	Eukaryotic cell lines
☒	☐	Palaeontology and archaeology
☒	☐	Animals and other organisms
☐	☒	Human research participants
☒	☐	Clinical data
☒	☐	Dual use research of concern

Methods

n/a	Involved in the study	
☒	☐	ChIP-seq
☒	☐	Flow cytometry
☒	☐	MRI-based neuroimaging

Human research participants

Policy information about studies involving human research participants

Population characteristics	See above
Recruitment	See above

Ethics oversight

All participants provided written consent, and the University of Western Ontario Ethics Review Board approved all data collection procedures.

Note that full information on the approval of the study protocol must also be provided in the manuscript.
